# Solitary Adrenal Metastasis from Esophageal Adenocarcinoma: A Case Report and Review of the Literature

**DOI:** 10.1155/2011/487875

**Published:** 2011-10-19

**Authors:** D. Dellaportas, P. Lykoudis, G. Gkiokas, G. Polymeneas, A. Kondi-Pafiti, D. Voros

**Affiliations:** ^1^2nd Department of Surgery, Aretaieion University Hospital, 115 28 Athens, Greece; ^2^1st Department of Pathology, Aretaieion University Hospital, 115 28 Athens, Greece

## Abstract

*Introduction*. In patients with extra-adrenal malignancy, an adrenal mass necessitates investigating the possibility of metastatic tumor. Curable adrenal metastasis are considered as a rare event. *Case report*. A 52-year-old male suffering from lower esophageal adenocarcinoma with a solitary left adrenal metastasis is presented herein, who underwent concomitant transhiatal esophagectomy and left adrenalectomy. The patient remains disease-free 18 months later. *Discussion*. Adrenal metastases mostly occur in patients with lung, kidney, breast, and gastrointestinal carcinomas. Primary esophageal adenocarcinoma gives adrenal metastatic deposits according to autopsy series with an incidence of about 3%–12%. When no other evidence of metastatic disease in cancer patients exists, several authors advocate adrenalectomy with curative intent. Isolated cases of long-term survival after resection of solitary adrenal metastasis from esophageal adenocarcinoma, like in our case, have been reported only as case reports. *Conclusion*. This study concludes that surgical resection may result in survival benefit in selected patients with solitary adrenal metastasis from esophageal adenocarcinoma.

## 1. Introduction

In patients with extra-adrenal malignancy, staging imaging modalities frequently reveal adrenal masses and require exclusion of the possibility of metastatic tumor spread [1]. Adrenal metastatic tumor usually indicates advanced malignancy and disseminated disease for most cancer patients, and especially for patients with primary esophageal cancer [2]. Rarely, radical surgical resection of the primary tumor associated with excision of the adrenal metastasis results in long-term survival. We present herein a case of a 52-year-old male suffering from esophageal adenocarcinoma with solitary metastasis to the left adrenal gland treated with concomitant transhiatal esophagectomy and left adrenalectomy. In his follow-up visits, he remains disease-free eighteen months later. A short review of the literature with reference to similar cases is also attempted.

## 2. Case report

**Figure 1:**
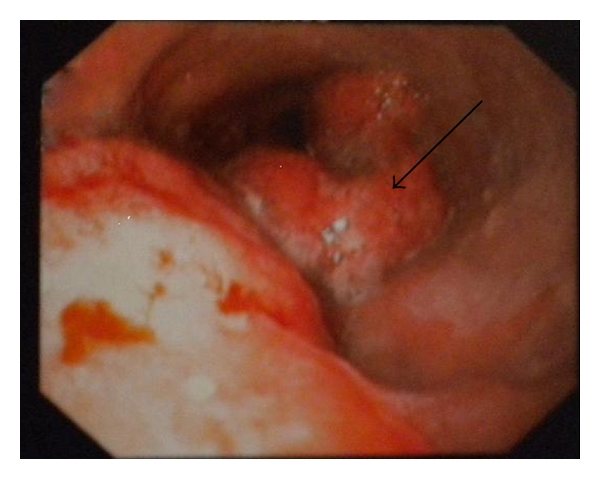
Endoscopy indicating a solid mass (arrow) narrowing the esophageal lumen.

**Figure 2:**
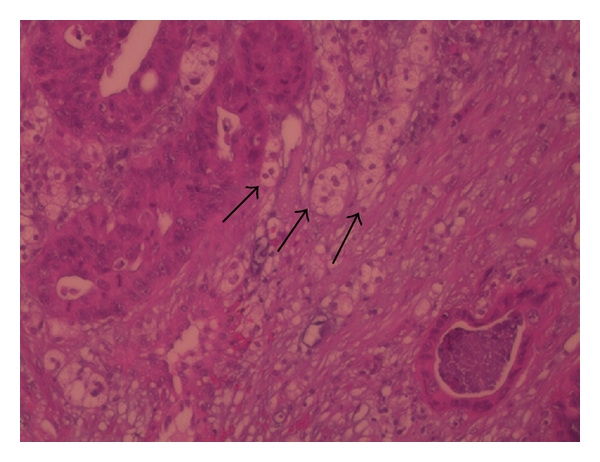
Histological section of the adrenal cortex showing extensive infiltration (arrows) by glandular structures (H-E, ×200).

A 52-year-old male presented complaining of dysphagia in both solid food and liquids for the last fifteen days. Esophagogastroduodenoscopy (EGD) revealed a solid mass narrowing the esophageal lumen on the lower third of the esophagus (Figure 1). Histopathological results, from EGD biopsy, demonstrated a moderately differentiated adenocarcinoma of the esophagus, growing on Barrett's esophagus background lesion. Staging computed tomography scan (CT-scan) of the thorax and the abdomen revealed a 2 × 2.5 cm mass lesion of the left adrenal gland, having suspicious imaging characteristics. Tumor markers were CEA 55 mlU/mL and Ca 19–9 854 IU/mL. The patient was fit for major surgery, and palliative operative approach was decided due to possible disseminated disease. The presence of a pheochromocytoma was excluded measuring urine vinyl-mandelic acid (VMA) levels, which were normal. Concomitant transhiatal esophagectomy and left adrenalectomy was performed, and an uneventful postoperative course followed. The patient was discharged on the 16th postoperative day, because of postoperative pneumonia, which was treated with intravenous antibiotics. Pathological examination of the esophagus confirmed the diagnosis and showed extensive adenocarcinoma infiltrating the esophageal wall and 4 out of 6 local lymph nodes positive for metastatic disease. The positive lymph nodes were paraesophageal 1 cm away from the primary lesion. Also, the adrenal gland was infiltrated by the same adenocarcinoma measuring 3 cm in diameter (Figure 2). In his follow-up visits, the patient remains disease-free eighteen months later.

## 3. Discussion

An adrenal mass lesion can represent multiple pathologic entities, benign or malignant. Most of these lesions are simple adenomas or secondary metastatic tumors [1]. Adrenal metastases mostly occur in patients with lung, kidney, breast, and gastrointestinal carcinomas, and rarely, melanoma or lymphoma and leuykemia involve the adrenals [3]. These lesions are frequently encountered during autopsy but uncommonly present clinically and are revealed during staging evaluation of a cancer patient using tomographic imaging modalities. Primary esophageal adenocarcinoma gives adrenal metastatic deposits quite frequently according to autopsy series with an incidence of about 3%–12% [3, 4], via hematogenous or lymphatic routes, and indicates disseminated disease. However, a solitary synchronous or metachronous metastasis for primary esophageal adenocarcinoma, like in our case, is a rare event, which confronts the physician to a therapeutic dilemma. In general, when no other evidence of metastatic disease in cancer patients exists, several authors advocate adrenalectomy with curative intent and provide evidence that aggressive surgical approach prolongs survival [5–7]. Although it is not an established principle in surgical oncology, concomitant primary tumor resection and adrenalectomy for metastatic disease has been reported for various cancer types in case reports [5–9].  Table 1 summarizes case reports similar to our case. More specifically, Fumagalli et al. analyzing 102 patients with esophagogastric adenocarcinoma found 5 patients suffering synchronous or metachronous adrenal metastasis and report better survival rates for surgical resection as long as the gland is the only site of metastasis beyond lymphonodal disease [10]. A Japanese study, also, reported long-term survival after resection for adrenal metastases from gastric cancer [11]. Also, isolated cases of long term survival after resection of solitary adrenal metastasis from esophageal adenocarcinoma, like in our case, have been reported only as case reports [12]. In our case, although the more conservative approach, regarding lymph-node dissection, of transhiatal esophagectomy was performed assuming microscopically disseminating disease, indicated by the adrenal metastasis, the patient remained disease free for long term interval. It is indicated from the above that in the absence of large case series for this rare clinical event, long-term disease-free survival even in case reports justifies aggressive surgical procedures in generally fit patients.

## 4. Conclusion

The management of a solitary adrenal metastasis in cancer patients can present as a surgical dilemma. This study concludes that surgical resection may result in survival benefit in selected patients with solitary adrenal metastasis from esophageal adenocarcinoma and should be considered as a therapeutic option. 

## Figures and Tables

**Table 1:** Adrenalectomy for metastatic disease in case reports.

Authors	Cancer primary site	No. of patients	Survival
Katayama et al. [8]	Colorectal	1	3 years and 5 months
Branum et al. [9]	Melanoma	8	Mean survival: 59 months
Fumagalli et al. [10]	Esophagogastric junction adenocarcinoma	5	3 long-term survivals
Mokuno et al. [11]	Gastric adenocarcinoma	1	40 months (bilateral adrenalectomy)
Saito et al. [12]	Esophageal adenocarcinoma	1	5 years and 11 months (he died of another reason)
